# LDAI-ISPS: LncRNA–Disease Associations Inference Based on Integrated Space Projection Scores

**DOI:** 10.3390/ijms21041508

**Published:** 2020-02-22

**Authors:** Yi Zhang, Min Chen, Ang Li, Xiaohui Cheng, Hong Jin, Yarong Liu

**Affiliations:** 1 School of Information Science and Engineering, Guilin University of Technology, Guilin 541004, China; zywait@glut.edu.cn (Y.Z.); hblyr@sina.com (Y.L.); 2 Hunan Institute of Technology, School of Computer Science and Technology, Hengyang 421002, China

**Keywords:** disease similarity, lncRNA similarity, space projection, computational prediction model

## Abstract

Long non-coding RNAs (long ncRNAs, lncRNAs) of all kinds have been implicated in a range of cell developmental processes and diseases, while they are not translated into proteins. Inferring diseases associated lncRNAs by computational methods can be helpful to understand the pathogenesis of diseases, but those current computational methods still have not achieved remarkable predictive performance: such as the inaccurate construction of similarity networks and inadequate numbers of known lncRNA–disease associations. In this research, we proposed a lncRNA–disease associations inference based on integrated space projection scores (LDAI-ISPS) composed of the following key steps: changing the Boolean network of known lncRNA–disease associations into the weighted networks via combining all the global information (e.g., disease semantic similarities, lncRNA functional similarities, and known lncRNA–disease associations); obtaining the space projection scores via vector projections of the weighted networks to form the final prediction scores without biases. The leave-one-out cross validation (LOOCV) results showed that, compared with other methods, LDAI-ISPS had a higher accuracy with area-under-the-curve (AUC) value of 0.9154 for inferring diseases, with AUC value of 0.8865 for inferring new lncRNAs (whose associations related to diseases are unknown), with AUC value of 0.7518 for inferring isolated diseases (whose associations related to lncRNAs are unknown). A case study also confirmed the predictive performance of LDAI-ISPS as a helper for traditional biological experiments in inferring the potential LncRNA–disease associations and isolated diseases.

## 1. Introduction

Long non-coding RNAs (LncRNAs) are a type of RNA, defined as being transcripts with lengths exceeding 200 nucleotides that are not translated into protein, which exist in all kinds of organisms widely [1,2]. A growing number of studies have found that mutations and dysregulations of lncRNAs cause a variety of diseases, including cervical cancer [3,4], colorectal cancer [5,6], ovarian cancer [7,8,9], prostate cancer [10,11], and diabetes [12,13]. Therefore, lncRNAs could be used as biomarkers for the early diagnosis and prognosis of corresponding cancers, which motivates the identification and confirmation of the associations between lncRNAs and diseases to become a research focus. The lncRNA related databases (such as LncRNAdb [14], LncRNADisease [15], NRED [16], and NONCODE [17]) provide strong data support on which the computational prediction models can be built to provide more accurate experimental targets as well as an effective supplement to biological experiments [18,19,20,21,22]:

- Provide guidance with less cost and time for the subsequent biological experimental verification related to complex diseases;
- Speed up our understanding on the pathogenesis of complex diseases;
- Give new ideas for disease prevention, diagnosis, treatment, and prognosis;
- Have a profound implication on drug development and medical improvement.

The computational models used for inferring lncRNA–disease associations have been divided into two main categories: machine learning-based inference and network-based inference.

In recent years, machine learning has been used to infer lncRNA–disease associations [23,24,25,26,27,28,29]. Zhao et al. [30] integrated multi-omic data, genomic, regulome, and transcriptome with naïve Bayesian classifier models to predict lncRNA–cancer associations, and successfully identified 707 potential cancer-related lncRNAs. Yu et al. [24] utilized the naïve Bayesian classifier to predict lncRNA–disease associations after constructing the global tripartite network and the global quadruple network. Lan et al. [31] used bagging support vector machine (SVM) to predict lncRNA–disease association based on multiple biological data resources fused by the matrix geometric mean. How to obtain the negative samples is the huge challenge that all of the above-mentioned machine learning based methods have to face. Normally, the unlabeled lncRNA–disease associations are those that cannot be found in a finite number of biological experiments, which are selected randomly to be the negative samples. Therefore, selecting the unknown associations as the negative samples randomly is unreasonable and will undoubtedly have a serious impact on the accuracy of prediction results, because it cannot mean that these associations do not exist. For overcoming that the negative sample cannot be obtained accurately, Chen et al. [32] proposed a semi-supervised learning framework (LRLSLDA) based on Laplacian Regularized Least Squares, without needing negative samples. LRLSLDA still has some defects, like needing too many parameters and low prediction accuracy. Considering that the accurate similarity network construction is beneficial to improve the prediction accuracy, Chen et al. [33] used the hyper geometric distribution to infer lncRNA–disease associations (HGLDA) without relying on known experimentally verified lncRNA–disease associations, but HGLDA cannot be used for isolated diseases and new lncRNAs.

The hypothesis that lncRNAs with similar functions tend to be related to similar diseases is the foundation of those network-based methods [34,35,36]. Chen et al. [37] proposed a new method named LNCSIM that only used the information of common ancestors to calculate the similarity without retaining the hierarchical structure of Directed Acyclic Graphs (DAGs) of diseases, which led to being more vulnerable to information bias in DAGs. Huang et al. [38] proposed an edge-based computational model ILNCSIM by integrating lncRNA–disease associations and disease DAGs. However, the prediction results of ILNCSIM were affected for lack of unlabeled but existing associations. Chen et al. [39] proposed a fuzzy measure-based lncRNA functional similarity calculation model (FMLNCSIM) that achieved better performance but still suffered from the information bias in DAGs. Inspired by social network analysis, Chen et al. [40] utilized Katz Centrality on network topology to predict lncRNA–disease associations. The predicted results were biased toward those diseases that had more related lncRNAs found from the known associations. Cheng et al. [41] proposed an integrative framework to predict novel lncRNA–disease associations, but the prediction result heavily relied on the integrated network and was easily affected by data incompleteness. Ding et al. [42] inferred the lncRNA–disease associations via lncRNA–disease–gene tripartite graph (TPGLDA), but TPGLDA depended on the topology of a tripartite graph whose data incompleteness may affect the predictive performance. Shi et al. [43] proposed a graph regression-based unified framework (GRUF) for the inference of lncRNA–disease associations including the inference of isolated diseases and new lncRNAs. GRUF provided more information on the relationships between a pair of lncRNA and disease instead of only a binary result. However, the prediction results may be affected by the quality of the dataset as well as those lncRNAs with low expression level. Numerous researchers introduced random walk into the prediction of lncRNA–disease associations [44,45,46,47,48,49,50,51,52,53,54]. Sun et al. [55] executed random walk with restart (RWR) on lncRNA functional similarity network to infer lncRNA–disease associations. Zhou et al. [56] implemented RWR on a heterogeneous network, which cannot avoid producing some biased predictions. Yao et al. [57] integrated genes, lncRNAs, phenotypes, and their associations into a multi-level composite network with which they implemented a RWR algorithm to identify candidate lncRNA–disease associations. Although this method can be used for isolated disease prediction, its prediction accuracy depended on the topology of the composite network and was affected by incompleteness and biases of data. The selection of a seed vector that was produced from known lncRNA–disease associations had a great influence on the predictive performance of the above-mentioned random walk algorithms. It caused the prediction results of the models that used the random walk algorithms to heavily depend on the known lncRNA–disease associations. Therefore, identifying diseases-related lncRNAs is still in its initial phase with following limitations:

- Most of the off-the-shelf computational models cannot be used for inferring isolated diseases and new lncRNAs directly;
- Supervised learning of machine learning needs a negative sample to train the class classifier, but such negative sample cannot be obtained;
- Those that only rely on the known network topology will produce biased prediction results.

Considering above limitations, we proposed a novel lncRNA–disease associations inference based on space projections of integrated networks (LDAI-ISPS) that contained the following four steps: step one, reconstruct the disease (lncRNA) integrated similarities network via integrating multiple network information; step two, change the Boolean network of known experimentally verified associations into the weighted network for further inferring the associations between lncRNAs and diseases accurately; step three, utilize the vector projections of the vectors coming from the networks of the above two steps to construct space projection scores; step four, obtain the final prediction results by integrating two kinds of space projection sores. Finally, LOOCV experiments and the case study showed that, without needing negative samples, LDAI-ISPS not only achieved excellent predictive performance, but also can be used for isolated diseases and new lncRNAs.

## 2. Results

### 2.1. Influence of Parameter Selection on Performance

**Figure 1:**
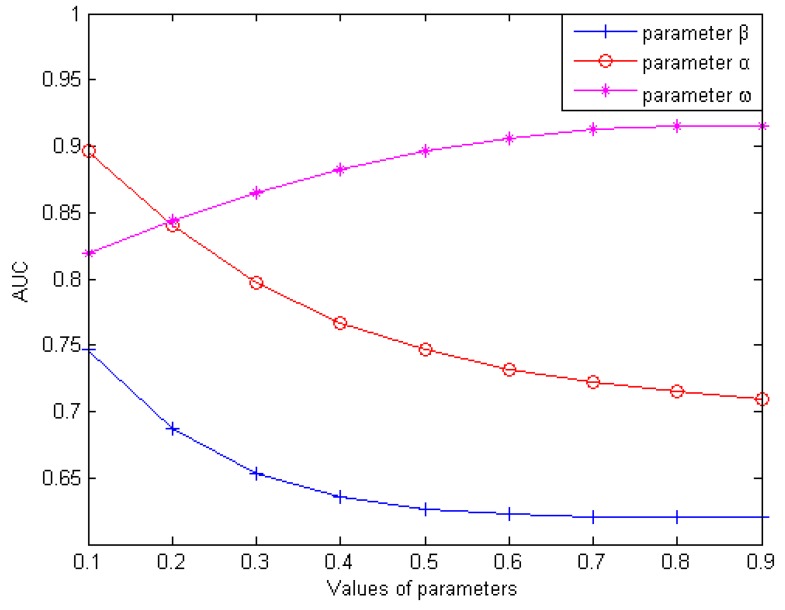
Influence of parameter variation on model prediction accuracy.

In this section, we mainly discuss how to obtain the optimum values that were the values when corresponding AUC values were highest with three weighting parameters (weighting parameter α used for the reconstruction of $LD_{nl\times nd}^{\left(dw\right)}$, weighting parameter β used for the reconstruction of $LD_{nl\times nd}^{\left(lw\right)}$, weighting parameter ω used for the integration of $LD_{nl\times nd}^{\left(pd\right)}$ and $LD_{nl\times nd}^{\left(pl\right)}$). Firstly, we analyzed how the value of β influenced the predictive validity of LDAI-ISPS with setting α and ω as 0.5 for simplicity. With increasing the value of β from 0.1 to 0.9 (with a step size of 0.1), we performed LOOCV on the dataset $LD_{nl\times nd}$ to calculate the AUC values (can be seen in Figure 1). We found that AUC was obtained the optimum value of 0.7463 when β was set to 0.1, and then AUC values decreased gradually. AUC obtained the minimum value of 0.6204 when β was set to 0.9. Secondly, after setting β to be 0.1 and ω to be 0.5, we performed LOOCV to observe the corresponding AUC values with increasing α from 0.1 to 0.9 (with step size of 0.1). Similarly, we found that AUC obtained the optimum value of 0.893915 when α was set to 0.1, and then AUC values decreased gradually. Based on above two steps (setting α=β=0.1), we performed LOOCV to calculate the corresponding AUC values with increasing ω from 0.1 to 0.9 (with step size of 0.1). We found that AUC obtained the optimum value of 0.9154 when ω was set to 0.8. In conclusion, we set α=β=0.1 and ω=0.8 to obtain the corresponding optimal AUC values.

### 2.2. Comparison with Other Methods

#### 2.2.1. Evaluation Metrics of Performance

LOOCV experiments were implemented for evaluating the predictive performance of LDAI-ISPS in inferring the latent lncRNA–disease associations. We divided the dataset into two parts. In one part, each known association in $LD_{nl\times nd}$ was observed in turn as a test data, and in the other part, the remaining known associations were used as the training data. Under the framework of LOOCV, we compared the prediction results with $LD_{nl\times nd}$ on some specific threshold to obtain the following four metrics: true positive (TP), false positive (FP), false negative (FN), true negative (TN). Furthermore, according to some specified thresholds, we calculated the true positive rate ($TPR=\frac{TP}{TP+FN}$) against false positive rate ($FPR=\frac{FP}{TN+FP}$) with which we plotted out the receiver operating characteristic curve (ROC). The area under the ROC curve (AUC) was finally calculated to assess the overall predictive performance of LDAI-ISPS.

#### 2.2.2. Comparison Results on Performance

**Figure 2:**
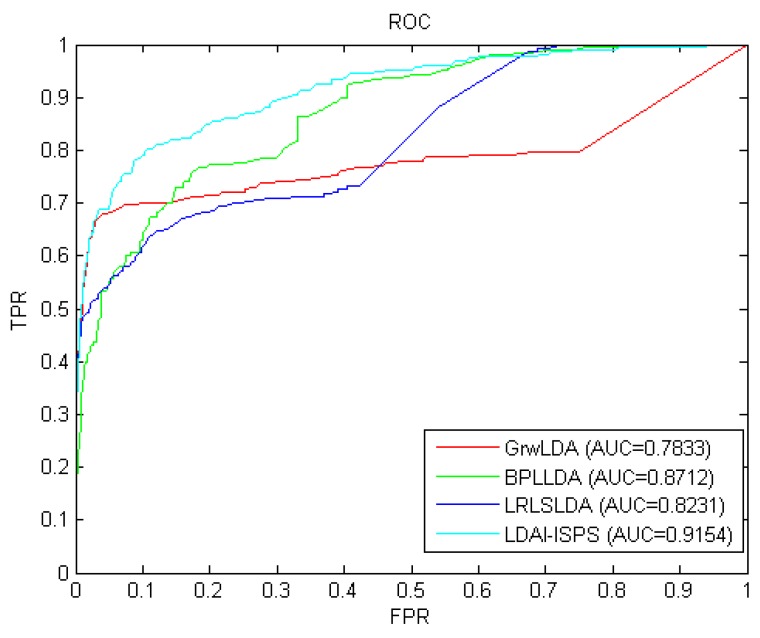
The receiver operating characteristic (ROC) curves and AUC values of the long non-coding RNA (lncRNA)–disease associations inference based on integrated space projection scores (LDAI-ISPS) compared with other methods.

Considering that the information used by GrwLDA [46], BPLLDA [58], and LRLSLDA [32] is similar to that of LDAI-ISPS and these four methods can all be used for isolated diseases and new lncRNAs, we compared LDAI-ISPS with GrwLDA, BPLLD, and LRLSLDA on predictive performance. In order to make an unbiased comparison, we used the same parameter values described in corresponding papers of GrwLDA, BPLLD, and LRLSLDA. The comparison results from LOOCV can be seen in Figure 2, where AUC values of GrwLDA, BPLLDA, LRLSLDA, and LDAI-ISPS were 0.7833, 0.8712, 0.8231, and 0.9154, respectively. Obviously, LDAI-ISPS obtained the optimum AUC value, which was higher than GrwLDA (16.84%), BPLLDA (5.07%), and LRLSLDA (11.21%).

### 2.3. Prediction for New lncRNAs and Isolated Diseases

New lncRNAs are those whose associations related to diseases are unknown. How to pair new lncRNAs with diseases remains an urgent challenge, and the solutions will certainly advance our understanding of disease molecular mechanisms. Firstly, although more and more new lncRNAs were discovered, their associations with diseases could not be identified by the time they were discovered. Secondly, no known association can be used directly to predict the potential associations. Thirdly, most of the existing computational methods cannot infer the potential associations between new lncRNAs and diseases. Therefore, we continuously removed all known related associations of each candidate lncRNA with diseases to simulate new lncRNAs and performed LOOCV to evaluate the predictive performance of LDAI-ISPS.

**Figure 3:**
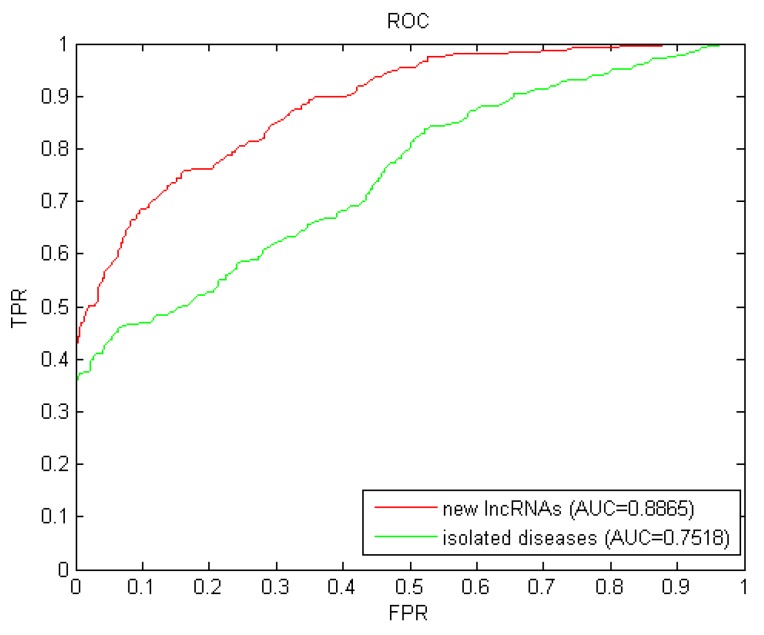
Results of LDAI-ISPS for new lncRNAs and isolated diseases.

Isolated diseases are those whose associations related to lncRNAs are unknown. The prediction for isolated diseases faced the similar challenge of new lncRNAs as mentioned before. We simulated each candidate disease as an isolated disease by removing all known related associations with lncRNAs, and then implemented LOOCV with these simulated isolated diseases. The AUC value of 0.7518 (shown in Figure 3) further confirmed the excellent predictive performance of LDAI-ISPS for isolated diseases.

### 2.4. Case Study

#### 2.4.1. Case Study for Potential Associations

Cervical cancer is a deadly threat to a woman’s life and health. It is estimated that there are 528,000 new cases and 266,000 deaths from cervical cancer worldwide every year [59]. Type 2 diabetes is one kind of metabolic disorder. So far, more than 415 million people have suffered terribly from type 2 diabetes. Therefore, identifying some specific diseases related lncRNAs is important for understanding the pathogenesis, treatment, and prognosis. We took cervical cancer and type 2 diabetes as the cases to further confirm the performance of LDAI-ISPS by using the known lncRNA-disease associations in $LD_{nl\times nd}$ as the training data.

The top five out of the prediction results for each of these two diseases are listed in Table 1, where two out of the top five predicted associations were found evidence in database LncRNADisease [15] and the remaining three out of the top five predicted associations were found supporting evidence in relevant literatures. Only one predicted association listed in Table 1 was not found any supporting evidence, which confirmed the excellent performance of LDAI-ISPS in inferring the potential associations between lncRNAs and diseases.

#### 2.4.2. Case Study for Isolated Diseases

We took prostate cancer and Alzheimer’s disease as the cases to further confirm the predictive performance of LDAI-ISPS for isolated diseases. As for prostate cancer, we removed 13 known associations related to it to simulate the isolated disease. As for Alzheimer’s disease, we removed eight known associations related to it to simulate the isolated disease. The top five out of the predicted results of both diseases were listed in Table 2, where all of the top five predicted associations related to prostate cancer were found evidence in database LncRNADisease, and four out of the top five predicted associations related to Alzheimer’s disease were found evidence in database LncRNADisease except CDKN2B-AS10. However, Tedde et al. [64] found that CDKN2A/CDKN2B genes/loci associated with late-onset Alzheimer’s disease, which proved the credibility of our LDAI-ISPS.

## 3. Discussion

The research of a computational model for inferring lncRNA–disease associations is still a hot topic. On one hand, the prediction of lncRNA–disease associations is helpful to explore the complex pathogenesis of diseases; on the other hand, the traditional biological methods are tedious and time-consuming, therefore, many computational methods have emerged in recent years used for inferring massive lncRNA–disease associations. Those computational methods still have some limitations that motivated us to propose a new lncRNA–disease association inference (LDAI-ISPS), whose main contribution consists of the following points: made full use of network topology characters instead of needing negative samples; LOOCV results showed that AUC value of LDAI-ISPS in inferring disease related lncRNAs was 0.9154, which was 16.86%, 5.07%, and 11.21% higher than that of GrwLDA, BPLLDA, and LRLSLDA, respectively. Additionally, the AUC value for new lncRNAs and isolated diseases were 0.8865 and 0.7518, respectively, which further evaluated the stronger predictive performance of LDAI-ISPS.

The following reasons helped our LDAI-ISPS achieve good predictive performance: first, we integrated Gaussian interaction profile central similarity to calculate disease similarity and lncRNA similarity, which made up the incompleteness of similarity network construction only with sematic similarity. Second, we reconstructed the Boolean network of known experimentally verified lncRNA–disease associations to be the weighted network of lncRNA–disease associations. The weighted network can exactly describe the association strength not just showing the existence of an association. Third, the global information (all the disease similarities, lncRNA similarities, and lncRNA–disease associations), even those diseases and lncRNAs without any known association, were utilized to improve the predictive ability of LDAI-ISPS including the ability on new lncRNAs and isolated diseases.

Our method still has following limitations that need to be improved in future: the predicted results were biased towards the diseases with more related lncRNAs or the lncRNAs with more related diseases; the prediction accuracy needs to be enhanced further through fusing different data. In conclusion, LDAI-ISPS can be a useful helper for inference of lncRNA–disease associations.

## 4. Materials and Methods

### 4.1. Materials

#### 4.1.1. LncRNA–Disease Association Network

The data source of our dataset used came from the LncRNADisease database [15] composed of experimentally supported lncRNA–disease association data and predicting novel lncRNA–disease associations. After preprocessing, the dataset was composed of 352 known associations that involved 156 lncRNAs (denoted by set $L=\{l_{1},l_{2},\ldots ,l_{nl}\}$) and 190 diseases (denoted by set $D=\{d_{1},d_{2},\ldots ,d_{nd}\}$), as shown in Supplementary Materials S1–S3. A Boolean matrix $LD_{nl\times nd}=(ld_{ij}{)}_{nl\times nd}$ including the 352 known lncRNA–disease associations was used to represent the adjacency matrix of the lncRNA–disease associations, where nl represented the number of lncRNAs with value of 156 and nd represented the number of diseases with value of 190. If lncRNA $l_{i}$ has a known association with disease $d_{j}$ checked from the 352 known lncRNA-disease associations, $ld_{ij}$ is set to 1, otherwise $ld_{ij}$ is set to 0.

#### 4.1.2. Disease Semantic Similarity

The method proposed by Wang et al. [65] defined the semantic contribution value of disease according to the layer structure of corresponding direct acyclic graph (DAG). A similar way $DAG(d_{j})=(N(d_{j}),E(d_{j}))$ was used as described in [65] to calculate the disease semantic similarity, where nodes set $N(d_{j})$ denoted the disease $d_{j}$ itself and its ancestors, and edges set $E(d_{j})$ denoted the relations between the nodes in DAG. For each node $d_{t}$ in set $N(d_{j})$, its contribution to $d_{j}$ was numerically defined as the following:

(1) $$C_{d_{j}}(d_{t})=\{\begin{array}{ll} 1, & \mathrm{if}\;d_{t}=d_{j}\\ \max\left\{\Delta *C_{d_{j}}(d_{t}^{\prime })|d_{t}^{\prime }\in \mathrm{children}\;\mathrm{of}\;d_{t}\;\right\}, & \mathrm{if}\;d_{t}\neq d_{j} \end{array}$$

where Δ is the contribution factor of connecting edges between $d_{t}$ and its children $d_{t}^{\prime }$, with the optimum value of 0.5 described in [65]. Additionally, the sematic score of disease $d_{j}$ was defined in Equation (2):

(2) $$SS(d_{j})=\sum_{d_{t}\in N(d_{j})}C_{d_{j}}(d_{t})$$

A matrix $DD_{nd\times nd}={\left(dd_{ij}\right)}_{nd\times nd}$ (can be seen in Supplementary Materials S4) represented the disease semantic similarities, where $dd_{ij}\in \left[0,1\right]$ denoted the semantic similarity between disease $d_{i}$ and $d_{j}$, with the calculation shown in Equation (3):

(3) $$dd_{ij}=\frac{\sum_{d_{t}\in N(d_{i})\cap N(d_{j})}C_{d_{i}}(d_{t})+C_{d_{j}}(d_{t})}{SS(d_{i})+SS(d_{j})}$$

#### 4.1.3. LncRNA Functional Similarity

**Figure 4:**
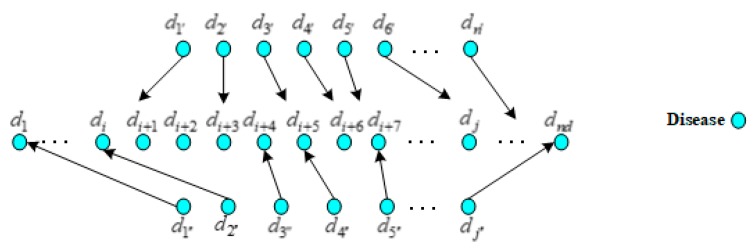
The mapping relations of the diseases associated with different lncRNAs.

By far, there have been many methods for lncRNA similarity network construction. We used the similar method like that proposed by Sun et al. [55], it calculated the functional similarity of paired lncRNAs by measuring the semantic similarity of diseases related to these two lncRNAs. As shown in Figure 4, it supposed that lncRNA $l_{i}$ and $l_{j}$ related to m diseases and n diseases out of 190 diseases, respectively, which formed the sets $D^{\left(l_{i}\right)}=\{d_{1^{\prime }},d_{2^{\prime }},\ldots ,d_{m^{\prime }}\}={\left\{d_{i^{\prime }}\right\}}_{m}\subset D$ and $D^{\left(l_{j}\right)}=\{d_{1^{\prime \prime }},d_{2^{\prime \prime }},\ldots ,d_{n^{\prime \prime }}\}={\left\{d_{j^{\prime \prime }}\right\}}_{n}\subset D$. The actual value of subscript $i^{\prime }$ ($j^{\prime \prime }$) was the subscript of each element in set $D^{\left(l_{i}\right)}$($D^{\left(l_{j}\right)}$) that was composed of m (n) diseases relating to lncRNA $l_{i}$ ($l_{j}$).

The similarity between one disease element $d_{i^{\prime }}$ in the set $D^{\left(l_{i}\right)}$ and the whole set $D^{\left(l_{i}\right)}$ was calculated as shown in Equation (4):

(4) $$S(d_{i^{\prime }},D^{\left(l_{i}\right)})=\underset{d_{t}\in D^{\left(l_{i}\right)}}{\max}(dd_{i^{\prime }t})$$

where the actual value of the subscripts $i^{\prime }$ and t were the subscripts of the corresponding diseases mapped by the diseases in set $D^{\left(l_{i}\right)}$, as mentioned before.

We defined a matrix $LL_{nl\times nl}={\left(ll_{ij}\right)}_{nl\times nl}$ (can be seen in Supplementary Materials S5), similar to the detailed calculation process described in ref. [55]. The functional similarities between lncRNAs were represented in matrix $LL_{nl\times nl}$, where $ll_{ij}\in [0,1]$ denoted the functional similarity between lncRNA $l_{i}$ and lncRNA $l_{j}$, with the calculation shown in Equation (5):

(5) $$ll_{ij}=\frac{\sum_{d_{t}\in D^{\left(l_{i}\right)}}S(d_{t},D^{\left(l_{i}\right)})+\sum_{d_{t}\in D^{\left(l_{j}\right)}}S(d_{t},D^{\left(l_{j}\right)})}{m+n}$$

where *m* and *n* refer the number of diseases related to lncRNA $l_{i}$ and lncRNA $l_{j}$ respectively.

### 4.2. Disease (LncRNA) Gaussian Interaction Profile Central Similarity

There are many zeros in matrix $DD_{nd\times nd}$ (and matrix $LL_{nl\times nl}$) because of few number of known associations as mentioned before; it means that the similarities related to these zeros between diseases (lncRNAs) cannot be found out from originally obtained information. Therefore, in order to further calculate the similarities between diseases (lncRNAs) accurately, the Gaussian interaction profile central similarities between lncRNAs were defined as $GL_{nl\times nl}={\left(gl_{ij}\right)}_{nl\times nl}$ with the calculation shown in Equation (6):

(6) $$gl_{ij}=\exp(-\gamma_{l}{\left\|LD(i,:)-LD(j,:)\right\|}^{2})$$

where $gl_{ij}$ denotes the Gaussian interaction profile central similarity between lncRNA $l_{i}$ and lncRNA $l_{j}$, LD(i,:) denotes the ith row of matrix $LD_{nl\times nd}$, the optimal value of parameter $\gamma_{l}$ that controlled the kernel bandwidth of Gaussian interaction profile in a similar way as described in ref. [66].

(7) $$\gamma_{l}=\frac{\gamma_{l}^{\prime }}{\frac{1}{nl}\sum_{i=1}^{nl}{\left\|LD(i,:)\right\|}^{2}}$$

where parameter $\gamma_{l}^{\prime }$ is set to 1 normally.

Similarly, the Gaussian interaction profile central similarity between diseases was defined as $GD_{nd\times nd}={\left(gd_{ij}\right)}_{nd\times nd}$ with the calculation shown in Equation (8):

(8) $$gd_{ij}=\exp(-\gamma_{d}{\left\|LD(:,i)-LD(:,j)\right\|}^{2})$$

where LD(:,i) denotes the ith column of matrix $LD_{nl\times nd}$, the optimal value of parameter $\gamma_{d}$ that controlled the kernel bandwidth was calculated as the following:

(9) $$\gamma_{d}=\frac{\gamma_{d}^{\prime }}{\frac{1}{nd}\sum_{i=1}^{nd}{\left\|LD(:,i)\right\|}^{2}}$$

where parameter $\gamma_{d}^{\prime }$ is set to 1 normally.

### 4.3. Disease (LncRNA) Integrated Similarities

Considering that only semantic similarity is not able to describe all the relationships between diseases accurately, we integrated disease semantic similarity and disease Gaussian interaction profile central similarity to further construct the disease integrated similarities network (denoted by $DD_{nd\times nd}^{\left(is\right)}={\left(dd_{ij}^{\left(is\right)}\right)}_{nd\times nd}$). It means that the Gaussian interaction profile central similarity is utilized to measure the similarity between two diseases when the corresponding element value in a disease semantic similarity matrix $DD_{nd\times nd}$ related to these two diseases is 0. Similarly, we integrated lncRNA functional similarity and lncRNA Gaussian interaction profile central similarity to construct the lncRNA integrated similarities network (denoted by $LL_{nl\times nl}^{\left(is\right)}={\left(ll_{ij}^{\left(is\right)}\right)}_{nl\times nl}$). The detailed formula description is shown as the following for clarity:

(10) $$dd_{ij}^{\left(is\right)}=\{\begin{array}{c} dd_{ij}\\ gd_{ij} \end{array}\begin{array}{c} ,\;\mathrm{if}\;dd_{ij}\neq 0\\ ,\;\mathrm{if}\;dd_{ij}=0 \end{array}$$

(11) $$ll_{ij}^{\left(is\right)}=\{\begin{array}{c} ll_{ij}\\ gl_{ij} \end{array}\begin{array}{c} ,\;\mathrm{if}\;ll_{ij}\neq 0\\ ,\;\mathrm{if}\;ll_{ij}=0 \end{array}$$

### 4.4. LDAI-ISPS Workflow Model

**Figure 5:**
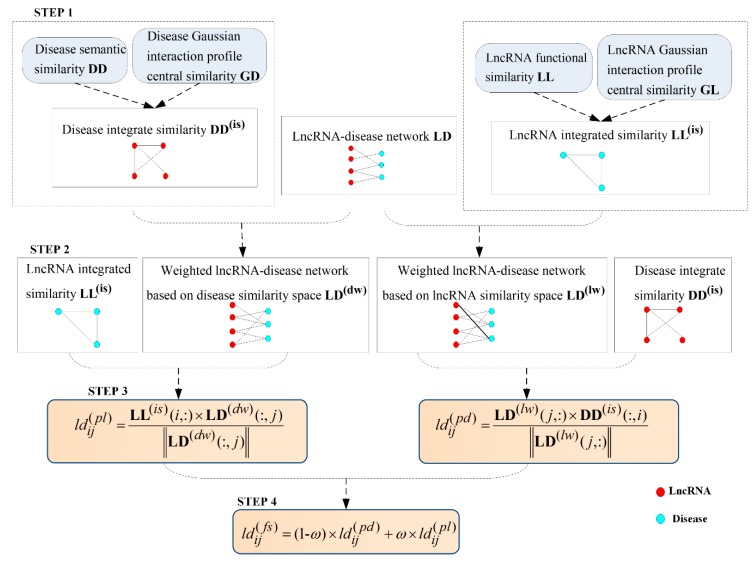
The flowchart of LDAI-ISPS.

After finishing the related data preparation, the detailed inferring process of LDAI-ISPS was explained in following flowchart (can be seen in Figure 5). Additionally, the numerical calculation results with each step are demonstrated in Supplementary Figure S1.

#### 4.4.1. Construction of lncRNA-Disease Weighted Network

Boolean network $LD_{nl\times nd}$ of known associations can only indicate whether the associations between lncRNAs and diseases exist, without showing the strength of associations. We utilized $LD_{nl\times nd}$, $DD_{nd\times nd}^{\left(is\right)}$, and $LL_{nl\times nl}^{\left(is\right)}$ to construct two weighted networks of lncRNA–disease associations (denoted by $LD_{nl\times nd}^{\left(dw\right)}={\left(ld_{ij}^{\left(dw\right)}\right)}_{nl\times nd}$ and $LD_{nl\times nd}^{\left(lw\right)}={\left(ld_{ij}^{\left(lw\right)}\right)}_{nl\times nd}$, respectively) for inferring the potential lncRNA–disease associations, with detailed calculation shown as the following:

(12) $$ld_{ij}^{\left(dw\right)}=ld_{ij}+\frac{\alpha \times \sum_{k=1,k\neq j}^{nd}dd_{kj}^{\left(is\right)}\times ld_{ik}}{sum(LD(i,:))}$$

where the weighting parameter α, used for the lncRNA–disease weighted network reconstruction based on disease semantic similarities, is set to [0, 1].

(13) $$ld_{ij}^{\left(lw\right)}=ld_{ji}+\frac{\beta \times \sum_{k=1,k\neq j}^{nl}ll_{jk}^{\left(is\right)}\times ld_{ki}}{sum(LD(:,i))}$$

where the weighting parameter β, used for the lncRNA–disease weighted network reconstruction based on lncRNA functional similarities, is set to [0, 1].

#### 4.4.2. Space Projection Scores of lncRNA–Disease Associations

Based on above two weighted lncRNA–disease networks ($LD_{nl\times nd}^{\left(dw\right)}$ and $LD_{nl\times nd}^{\left(lw\right)}$), we defined the space projection scores of lncRNA–disease associations (denoted by $LD_{nl\times nd}^{\left(pd\right)}={\left(ld_{ij}^{\left(pd\right)}\right)}_{nl\times nd}$ and $LD_{nl\times nd}^{\left(pl\right)}={\left(ld_{ij}^{\left(pl\right)}\right)}_{nl\times nd}$, respectively) by utilizing the concept of vector projection to infer the associations between lncRNAs and diseases more accurately.

(14) $$ld_{ij}^{\left(pd\right)}=\frac{LD^{\left(lw\right)}(j,:)\times DD^{\left(is\right)}(:,i)}{\left\|LD^{\left(lw\right)}(j,:)\right\|}$$

where $ld_{ij}^{\left(pd\right)}$ indicates one of the space projection scores obtained from the disease similarity network $DD_{nd\times nd}^{\left(is\right)}$, and $\left\|LD^{\left(lw\right)}(j,:)\right\|$ is the 2-norm based on row vector $LD^{\left(lw\right)}(j,:)$.

(15) $$ld_{ij}^{\left(pl\right)}=\frac{LL^{\left(is\right)}(i,:)\times LD^{\left(dw\right)}(:,j)}{\left\|LD^{\left(dw\right)}(:,j)\right\|}$$

where $ld_{ij}^{\left(pl\right)}$ indicates one of the space projection scores obtained from the lncRNA integrated similarities network $LL_{nl\times nl}^{\left(is\right)}$, and $\left\|LD^{\left(dw\right)}(:,j)\right\|$ is the 2-norm based on column vector $LD^{\left(dw\right)}(:,j)$.

#### 4.4.3. Prediction Score Based on Space Projection Scores

The final prediction score of lncRNA–disease associations (denoted by $LD_{nl\times nd}^{\left(fs\right)}={\left(ld_{ij}^{\left(fs\right)}\right)}_{nl\times nd}$) was composed of two parts, space projection score $LD_{nl\times nd}^{\left(pd\right)}$ and space projection score $LD_{nl\times nd}^{\left(pl\right)}$:

(16) $$ld_{ij}^{\left(fs\right)}=(1-\omega )\times ld_{ij}^{\left(pd\right)}+\omega \times ld_{ij}^{\left(pl\right)}$$

where the weighting parameter ω that represents the importance degree of $ld_{ij}^{\left(pl\right)}$ is set to [0, 1], the larger value of $ld_{ij}^{\left(fs\right)}$ means the greater probability of an association existing between lncRNA $l_{i}$ and diseases $d_{j}$.

## Figures and Tables

**Table 1:** The top five results predicted for cervical cancer and type 2 diabetes.

Disease	lncRNA Name	Evidence	Rank
Cervical cancer	LSINCT5	Ref. [60]	1
Cervical cancer	HOTAIR	LncRNADisease	2
Cervical cancer	MEG3	LncRNADisease	3
Cervical cancer	EPB41L4A-AS1	Ref. [61]	4
Cervical cancer	PANDAR	Ref. [3]	5
Type 2 diabetes	IGF2-AS	Ref. [62]	1
Type 2 diabetes	MEG3	LncRNADisease	2
Type 2 diabetes	PINK1-AS	Ref. [63]	3
Type 2 diabetes	Gas5	LncRNADisease	4
Type 2 diabetes	PCAT-1	Unconfirmed	5

**Table 2:** The top five results predicted for specific isolated diseases (e.g., prostate cancer and Alzheimer’s disease).

Disease	lncRNA Name	Evidence	Rank
Prostate cancer	PCAT-1	LncRNADisease	1
Prostate cancer	C1QTNF9B-AS1	LncRNADisease	2
Prostate cancer	CBR3-AS1	LncRNADisease	3
Prostate cancer	PCA3	LncRNADisease	4
Prostate cancer	PCAT1	LncRNADisease	5
Alzheimer’s disease	BACE1-AS	LncRNADisease	1
Alzheimer’s disease	GDNFOS	LncRNADisease	2
Alzheimer’s disease	SNHG3	LncRNADisease	3
Alzheimer’s disease	SOX2-OT	LncRNADisease	4
Alzheimer’s disease	CDKN2B-AS10	Ref. [64]	5

## Supplementary Materials

The following are available online at https://www.mdpi.com/1422-0067/21/4/1508/s1, Figure S1: Numerical calculation results of LDAI-ISPS workflow model, Table S1: Known lncRNA-disease associations, Table S2: LncRNA names, Table S3: Disease names, Table S4: Disease semantic similarity matrix, Table S5: LncRNA functional similarity matrix.

## Abbreviations

LOOCV	leave-one-out cross validation
ROC	receiver operating characteristic
AUC	area under the ROC curve
FPR	false positive rate
TPR	true positive rate
