# Supplementary figures and images for: LDAI-ISPS: LncRNA–Disease Associations Inference Based on Integrated Space Projection Scores
Source: Int J Mol Sci. 2020 Feb 22;21(4):1508. doi: 10.3390/ijms21041508 (PMC7073162; doi:10.3390/ijms21041508)

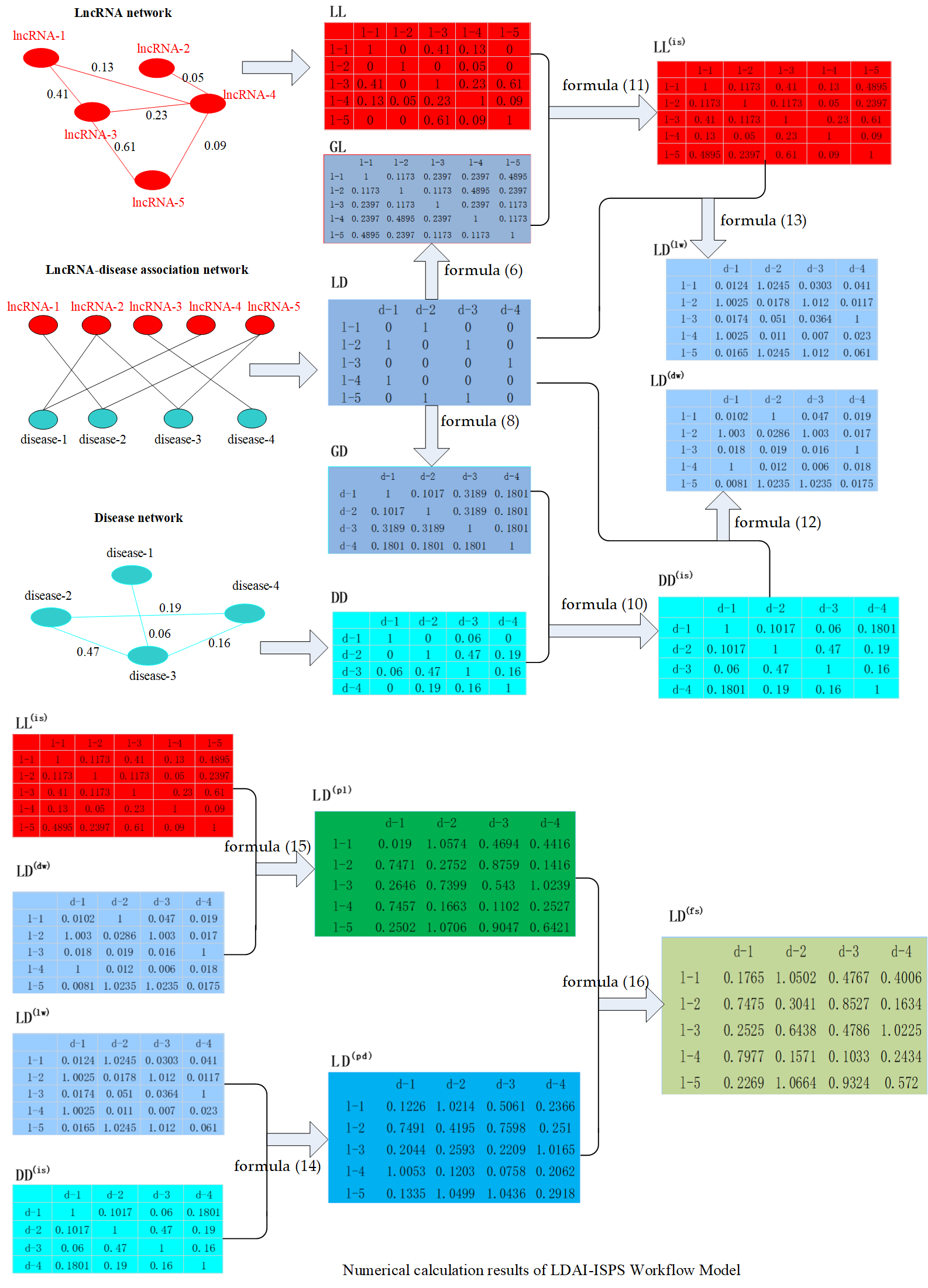

Supplement: Supplementary file 1 [file ijms-21-01508-s001.zip › Manuscriptand Supplementary Materials/Supplementary Figure 1.tif]
